# A comprehensive analysis of patients with cerebral arteriovenous malformation with headache: assessment of risk factors and treatment effectiveness

**DOI:** 10.1186/s10194-024-01774-7

**Published:** 2024-05-07

**Authors:** Haibin Zhang, Heze Han, Li Ma, Ruinan Li, Zhipeng Li, Anqi Li, Kexin Yuan, Qinghui Zhu, Chengzhuo Wang, Yukun Zhang, Hongwei Zhang, Dezhi Gao, Geng Guo, Shuai Kang, Xun Ye, Youxiang Li, Shibin Sun, Hao Wang, Qiang Hao, Yu Chen, Rong Wang, Xiaolin Chen, Yuanli Zhao, Registry of Multimodality Treatment for Brain Arteriovenous Malformation in Mainland China (MATCH)

**Affiliations:** 1 Department of Neurosurgery, Beijing Tiantan Hospital, Capital Medical University, Beijing, China; 2 Center for Cerebrovascular Research, University of California, San Francisco, CA USA; 3 Department of Neurosurgery, Peking University International Hospital, Peking University, Beijing, China; 4 Department of Gamma-Knife Center, Beijing Tiantan Hospital, Capital Medical University, Beijing, China; 5 Department of Emergency, First Hospital of Shanxi Medical University, Taiyuan, Shanxi Province China; 6 Department of Interventional Neuroradiology, Beijing Tiantan Hospital, Beijing, China; 7 China National Clinical Research Center for Neurological Diseases, Beijing, China; 8 Beijing Neurosurgical Institute, Beijing Tiantan Hospital, Capital Medical University, Beijing, China; 9 Department of Neurosurgery, Peking Union Medical College Hospital, Chinese Academy of Medical Sciences and Peking Union Medical College, Beijing, China

**Keywords:** Arteriovenous malformation, Headache, Intervention treatments, Conservative treatment

## Abstract

**Background:**

Due to the high mortality and disability rate of intracranial hemorrhage, headache is not the main focus of research on cerebral arteriovenous malformation (AVM), so research on headaches in AVM is still scarce, and the clinical understanding is shallow. This study aims to delineate the risk factors associated with headaches in AVM and to compare the effectiveness of various intervention treatments versus conservative treatment in alleviating headache symptoms.

**Methods:**

This study conducted a retrospective analysis of AVMs who were treated in our institution from August 2011 to December 2021. Multivariable logistic regression analysis was employed to assess the risk factors for headaches in AVMs with unruptured, non-epileptic. Additionally, the effectiveness of different intervention treatments compared to conservative management in alleviating headaches was evaluated through propensity score matching (PSM).

**Results:**

A total of 946 patients were included in the analysis of risk factors for headaches. Multivariate logistic regression analysis identified that female (OR 1.532, 95% CI 1.173–2.001, *p* = 0.002), supply artery dilatation (OR 1.423, 95% CI 1.082–1.872, *p* = 0.012), and occipital lobe (OR 1.785, 95% CI 1.307–2.439, *p* < 0.001) as independent risk factors for the occurrence of headaches. There were 443 AVMs with headache symptoms. After propensity score matching, the microsurgery group (OR 7.27, 95% CI 2.82–18.7 *p* < 0.001), stereotactic radiosurgery group(OR 9.46, 95% CI 2.26–39.6, *p* = 0.002), and multimodality treatment group (OR 8.34 95% CI 2.87–24.3, *p* < 0.001) demonstrate significant headache relief compared to the conservative group. However, there was no significant difference between the embolization group (OR 2.24 95% CI 0.88–5.69, *p* = 0.091) and the conservative group.

**Conclusions:**

This study identified potential risk factors for headaches in AVMs and found that microsurgery, stereotactic radiosurgery, and multimodal therapy had significant benefits in headache relief compared to conservative treatment. These findings provide important guidance for clinicians when developing treatment options that can help improve overall treatment outcomes and quality of life for patients.

**Supplementary Information:**

The online version contains supplementary material available at 10.1186/s10194-024-01774-7.

## Introduction

Cerebral arteriovenous malformations (AVM) are typically considered congenital central nervous system vascular anomalies, characterized by abnormal direct shunting between arteries and veins, and the absence of a normal capillary network [1]. The main clinical manifestations were intracranial hemorrhage, epilepsy, and headache. It is reported that about 5% to 14% of AVM patients (AVMs) experience headaches [2]. Although the incidence of headaches is relatively low, they still occupy a large proportion of the population, and headache often affects the daily life of patients and reduce their quality of life [3]. It is important to understand the risk factors and treatment effects of headaches in AVM for developing effective management strategies.

Due to the high mortality and disability rate of intracranial hemorrhage, [4] headache is not the primary focus of cerebral AVMs, so the study of headache in AVM is still scarce, and the clinical understanding is shallow [5]. Prior studies have suggested that the absence of stenosis in the draining vein is a potential risk factor for headaches in unruptured AVMs [6]. However, due to limitations such as unclear headache types, small sample size, and no statistical correction, this conclusion needs to be further verified and extended in a larger database.

Additionally, the management of AVM primarily focuses on preventing and minimizing the risk of intracranial hemorrhage. This can be achieved through various treatments, including microsurgical resection, stereotactic radiosurgery (SRS), endovascular embolization, and multimodality treatment [7]. Each intervention treatment possesses distinct advantages and limitations [8]. However, there is a notable paucity of attention towards the relative efficacy of these interventions in alleviating headache symptoms. Consequently, the current understanding of headache outcomes remains inadequately explored.

Therefore, this study aims to delineate the risk factors associated with headaches in AVM and to compare the effectiveness of various intervention treatments versus conservative treatment in alleviating headache symptoms. By focusing on and comprehensively understanding this often-overlooked aspect of AVM research, devising rational and effective management strategies for patients with headaches is crucial for enhancing their quality of life.

## Methods

### Study design and participants

This retrospective observational study aimed to investigate the risk factors associated with headaches in AVMs and assess the effectiveness of different headache treatments. The study utilized data from a single-center database, which was prospectively obtained through the MATCH Registry sponsored by Beijing Tiantan Hospital. The MATCH study, registered on ClinicalTrials.gov under NCT04572568, was a nationwide multicenter prospective collaboration registry designed to explore the natural history of AVMs in Asia and identify optimal management strategies for these conditions [9]. Further details regarding the comprehensive protocol for data quality management in the MATCH registry can be found in Supplemental Material 1.

**Fig. 1:**
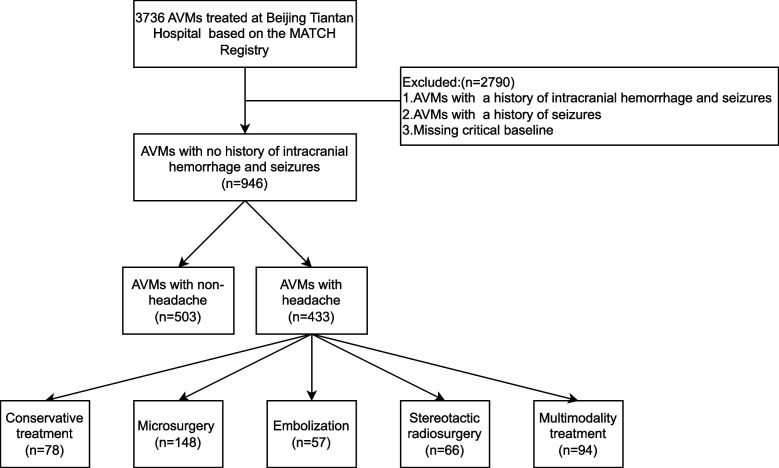
Patient flowchart

From August 2011 to December 2021, a continuous recruitment process yielded a total of 3736 AVMs at our institution. A meticulous analysis of medical records and imaging data was conducted, leading to the exclusion of certain patients based on specific criteria. We excluded the following patients:1) lack of key clinical baseline data. 2) Patients with a history of intracranial hemorrhage.3)Patients with a history of seizures. Subsequently, 946 AVMs were included in the analysis to identify risk factors associated with headaches. In terms of treatment modalities, the 443 patients with headaches were categorized into the conservative treatment group, microsurgery group, embolization group, stereotactic radiosurgery group, and multimodality treatment group, as illustrated in Fig. 1. This classification allowed for the evaluation of the impact of different treatments on headache outcomes.

The Institutional Review Board of Beijing Tiantan Hospital approved this study (KY 2020–003-01). Written informed consent for collecting clinical information was obtained at admission. All the analyses were carried out according to the Helsinki Declaration guideline. This study was reported in accordance with the STROBE guidelines for observational studies.

### Baseline characteristics

Demographic factors (age, sex), morphological, vascular structural characteristics, AVM location, Spetzler-Martin grade, and headache details were systematically recorded as potential confounders. The terminology used for morphological and angioarchitecture characteristics adhered to the definitions provided by the joint committee led by the American Society of Interventional and Therapeutic Neuroradiology. This encompassed features like feeding artery dilation, perforating feeding artery, associated aneurysm, draining vein stenosis, deep drainage vein, and venous aneurysm [10].

All radiological characteristics were independently evaluated by two credentialed senior neurointerventional radiologists. If inconsistency was present, the final determination was made by a senior professor of neurointerventional radiology with more than 30 years of clinical experience.The definition of the eloquent area aligned with the evaluation criteria in the Spetzler-Martin (SM) Grading system and previous neuroanatomy-function mapping [11]. Deep location was defined as any location involving the brainstem, cerebellum, basal ganglia, thalamus, corpus callosum, or insular lobe [12].

Information about headache characteristics was extracted from patient medical records and follow-up interviews. According to the International Classification of Headache Disorders, attributing a headache to an AVM only requires: (1) the headache led to the discovery of the AVM or the headache improves/worsens depending on the course of the AVM, (2) the headache localizes to the AVM site, and (3) the headache is not attributable to another cause including intracerebral hemorrhage [13]. Headaches were categorized by frequency into occasional, frequent, and chronic. Occasional headache was defined as frequency of headache onset < 1 day/month (< 12 days per year). Frequent headache was defined as frequency of headache onset 1–14 days/month (≥ 12 days and < 180 days per year). Chronic headache was defined as the frequency of headache onset ≥ 15 days/month, more than 3 months(≥ 180 days per year) [13]. Mild headache was defined as mild discomfort in the head that did not disrupt daily life. Moderate headache was defined as significant pain in the head that, while tolerable, interfered with the patient's daily activities. Severe headache was defined as severe pain in the head that was intolerable and significantly interfered with the patient's daily activities.

### Outcomes and follow-up

In the outcome evaluation of this study, our focus centered on determining whether patients experienced relief of headache symptoms after receiving intervention or conservative treatment. A favorable outcome event was defined as the complete or significant alleviation of headache symptoms during the follow-up period. Conversely, an unfavorable outcome event was defined as patients whose headache symptoms either worsened remained unchanged, or showed only mild relief throughout the follow-up.

Outcomes were assessed via phone interviews or record review by well-trained clinical research coordinators at 3 months, annually (1, 2, and 3 years), and every 5 years after the treatment decision. The inception point of the follow-up was the date of clinical presentation onset that led to the diagnosis of the AVM for the conservative treatment group, and the date of receipt of the intervention for the interventional groups. The endpoint was the date of the patient's last follow-up or death.

### Controlling for confounding

To minimize the impacts of potential confounding and selection bias in outcome comparisons between intervention and conservative treatment for AVM headache, propensity score matching (PSM) was used to compensate for differences in baseline characteristics between the conservative treatment group and intervention treatment group (microsurgery group, embolization group, stereotactic radiosurgery group, and multimodality treatment group). A propensity score was calculated using logistic regression, and 1:1 patient matching with a caliper of 0.1 standard deviation was performed using the nearest-neighbor matching method without replacement. Baseline characteristics, including demographic factors, morphological, vascular structural characteristics, AVM location, and headache characteristics were matched between the conservative treatment group and intervention treatment group.

### Statistical analysis

Categorical variables were presented as counts (with percentages), while continuous variables were presented as mean ± SD, in the case of normal distribution, or median and interquartile range (IQR), for non-normal distribution. Pearson chi-square test or Fisher exact test was used to compare categorical variables as appropriate. After testing for normality, continuous variables were analyzed using the independent Student t-test or Mann–Whitney U rank-sum test as appropriate.

Univariate and multivariate Logistic regression analyses were used to estimate the odds ratio and 95% confidence intervals for potential risk factors for AVM headache. Variables with *p* < 0.05 in the univariate analysis were included in the multivariate regression analysis. Univariate and multivariate logistic regression analysis was used to compare the odds ratio and 95% confidence interval for headache outcomes between the conservative treatment group and the intervention treatment group.

Statistical analyses of the data were performed using SPSS software (version 26.0, IBM Corp). Statistical significance was set at *P* < 0.05 (2-sided).

### Data availability

The data that support the findings of this study are available from the corresponding author upon reasonable request.

## Results

### Patient demographics and characteristics between AVMs with headache and non-headache

A total of 946 patients were included in the analysis of risk factors for headaches. The median age at diagnosis was 29.44 years, with 42.18% of patients being female. Patients were categorized into headache and non-headache groups, and their characteristics were summarized in Table 1.

**Table 1:** Baseline characteristics between AVMs with headache and non-headache

Variable	Overall	No Headache	Headache	*P*-value
	(*n* = 946)	(*n* = 503)	(*n* = 443)	
Age, median,(IQR), yrs	29.44 (17.14,40.53)	31.19 (16.51,42.54)	27.71 (17.66,37.85)	0.06
Female	399 (42.18%)	188 (37.38%)	211 (47.63%)	< 0.01*
Feeding artery				
Dilation	566 (59.83%)	272 (54.08%)	294 (66.37%)	< 0.01*
Perforating artery	314 (33.19%)	183 (36.38%)	131 (29.57%)	0.03*
Associated aneurysm	149 (15.75%)	81 (16.10%)	68 (15.35%)	0.75
Diffuse nidus	232 (24.52%)	123 (24.45%)	109 (24.60%)	0.96
Draining veins				
Stenosis	118 (12.47%)	66 (13.12%)	52 (11.74%)	0.52
Any deep drainage	346 (36.58%)	199 (39.56%)	147 (33.18%)	0.04*
Venous aneurysm	270 (28.54%)	136 (27.04%)	134 (30.25%)	0.28
AVM largest diameter(mm)	35.83 (25.00,48.80)	35.00 (23.77,48.06)	37.37 (26.31,49.78)	0.04*
AVM location				
Frontal	227 (24.00%)	119 (23.66%)	108 (24.38%)	0.80
Temporal	229 (24.21%)	126 (25.05%)	103 (23.25%)	0.52
Parietal	249 (26.32%)	124 (24.65%)	125 (28.22%)	0.21
Occipital	265 (28.01%)	106 (21.07%)	159 (35.89%)	< 0.01*
Subtentorial location	123 (13.00%)	84 (16.70%)	39 (8.80%)	< 0.01*
Eloquent location	541 (57.19%)	299 (59.44%)	242 (54.63%)	0.14
Deep location	274 (28.96%)	177 (35.19%)	97 (21.90%)	< 0.01*
Spetzler-Martin grade				0.40
I	125 (13.21%)	70 (13.92%)	55 (12.42%)	
II	294 (31.08%)	151 (30.02%)	143 (32.28%)	
III	327 (34.57%)	165 (32.80%)	162 (36.57%)	
IV	139 (14.69%)	81 (16.10%)	58 (13.09%)	
V	61 (6.45%)	36 (7.16%)	25 (5.64%)	

*IQR* Interquartile range, *AVM* Cerebral arteriovenous malformations, *NA* Not applicable

^*^Statistical significance (*p* < 0.05)

Among the patients, 59.83% had dilated supplying arteries, 33.19% were fed by perforating arteries, and 36.58% were drained by deep veins. The median size of the AVM nidus (maximum diameter) was 35.83 mm. Notably, 28.1% of the nidus were located in the occipital lobe, 28.96% in deep locations, 57.19% in eloquent locations, and 13.00% in the subtentorial location. Significant differences were observed between the headache and non-headache groups regarding female, dilation, perforating artery, any deep drainage, AVM largest diameter, occipital location, subtentorial location, and deep location.

Of the 443 AVMs associated with headaches, 37.92% had mild headaches, 51.02% had moderate headaches, and 11.06% had severe headaches. Most patients experienced occasional headaches, with 22.35% of AVMs causing frequent headaches, and only 2.93% resulting in chronic headaches. In addition, 11.74% of patients had an identifiable headache trigger, which was usually triggered when they were agitated or overworked. About 4.97% of the patients experienced aura symptoms, primarily visual disturbances before the onset of headaches.38.83% of patients used painkillers to relieve symptoms. Throbbin and dull pain were the most common descriptions of the properties of pain (Table 2).

**Table 2:** Headache characteristics in AVMs

Variable	Overall (*n* = 443)
Headache severity	
Mild	168 (37.92%)
Moderate	226 (51.02%)
Severe	49 (11.06%)
Headache frequency	
Occasional	331 (74.72%)
Frequent	99 (22.35%)
Chronic	13 (2.93%)
Existence inducement	
Yes	52 (11.74%)
No	356 (80.36%)
Missing	35 (7.90%)
Aura symptoms	
Yes	22 (4.97%)
No	385 (86.91%)
Missing	36 (8.13%)
Headache relief methods	
Spontaneous remission	232 (52.37%)
Take painkillers	172 (38.83%)
Missing	39 (8.80%)
Description of pain properties	
Throbbing pain	171 (38.6%)
Dull pain	52 (11.74%)
Sharp pain	31 (7.00%)
Electric shock pain	5 (1.13%)
Missing	184 (41.53%)
Accompanied by nausea or vomiting	
Yes	122 (27.54%)
No	290 (65.46%)
Missing	31 (7.00%)

*AVMs* Cerebral arteriovenous malformations patients

### Risk factors associated with headaches in AVMs

In a univariate logistic regression analysis, female (OR 1.524, 95% CI 1.175–1.976, *p* = 0.001), supply artery dilatation (OR 1.676, 95% CI 1.287–2.182, *p* < 0.001), and occipital lobe (OR 2.097, 95% CI 1.570–2.800, *p* < 0.001) were found to be significantly associated with an increased risk of headache in AVMs. Conversely, perforating arteries (OR 0.734, 95% CI 0.559–0.965, *p* = 0.027), any deep drainage (OR 0.759, 95% CI 0.581–0.990, *p* = 0.042), subtentorial location (OR 0.482, 95% CI 0.322–0.721, *p* < 0.001), and deep location (OR 0.516, 95% CI 0.386–0.690, *p* < 0.001) were identified as factors that reduced the risk of headaches in AVMs. Subsequently, we conducted a multivariate logistic regression analysis including the aforementioned seven related factors and ultimately demonstrated that female (OR 1.532, 95% CI 1.173–2.001, *p* = 0.002), supply artery dilatation (OR 1.423, 95% CI 1.082–1.872, *p* = 0.012), and occipital lobe (OR 1.785, 95% CI 1.307–2.439, *p* < 0.001) independently predicted the occurrence of headaches (Table 3).

**Table 3:** Univariate and multivariate logistic analysis of risk factors for headache in AVM

Characteristics	Univariate analysis			Multivariate analysis		
	OR	95%CI	*P*-value	OR	95%CI	*P*-value
Age	0.991	(0.983,1)	0.053			
Female	1.524	(1.175,1.976)	0.001*	1.532	(1.173,2.001)	0.002*
Feeding artery						
Dilation	1.676	(1.287,2.182)	< 0.001*	1.423	(1.082,1.872)	0.012*
Perforating artery	0.734	(0.559,0.965)	0.027*	0.853	(0.613,1.186)	0.344
Associated aneurysm	0.945	(0.665,1.342)	0.751			
Diffuse nidus	1.008	(0.749,1.357)	0.957			
Draining veins						
Stenosis	0.881	(0.597,1.298)	0.521			
Any deep drainage	0.759	(0.581,0.990)	0.042*	0.886	(0.635,1.236)	0.475
Venous aneurysm	1.170	(0.882,1.552)	0.275			
AVM largest diameter(mm)	1.006	(1.000,1.013)	0.057			
AVM location						
Frontal	1.040	(0.772,1.403)	0.796			
Temporal	0.906	(0.672,1.222)	0.519			
Parietal	1.201	(0.899,1.605)	0.214			
Occipital	2.097	(1.570,2.800)	< 0.001*	1.785	(1.307,2.439)	< 0.001*
Subtentorial location	0.482	(0.322,0.721)	< 0.001*	0.715	(0.427,1.197)	0.202
Eloquent location	0.821	(0.635,1.063)	0.135			
Deep location	0.516	(0.386,0.690)	< 0.001*	0.845	(0.550,1.298)	0.442

*AVM* Cerebral arteriovenous malformations, *CI* Confidence interval, *OR* Odds Ratio

^*^Statistical significance (*p* < 0.05)

### Headache outcome between the conservative treatment group and intervention treatment group

Before propensity score matching (PSM), the baseline characteristics of patients in each treatment group were presented in Table 4. The median follow-up time was 6.63(4.00–9.85) years for the conservative treatment group and 5.63(3.19–8.50) years for the intervention groups. Headache relief was significantly observed in 50% of patients receiving conservative treatment, 92.57% of patients undergoing microsurgery, 75.44% of patients treated with embolization, 92.42% of patients who underwent stereotactic radiosurgery, and 86.17% of patients receiving multimodality treatment (Table 5). Compared to the conservative group, intervention treatments resulted in significant headache relief.

**Table 4:** Baseline characteristics between conservative treatment group and intervention treatment group

Variable	Conservative treatment (*n* = 78)	Microsurgery (*n* = 148)	Embolization (*n* = 57)	Stereotactic radiosurgery (*n* = 66)	Multimodality treatment (*n* = 94)
Age, median,(IQR), yrs	28.18 (18.86,39.05)	27.10 (16.99,37.88)	28.64 (20.30,35.71)	27.71 (16.87,39.71)	29.59 (18.70,37.42)
Female	36 (46.15%)	59 (39.86%)	35 (61.4%)	36 (54.55%)	45 (47.87%)
Feeding artery					
Dilation	53 (67.95%)	98 (66.22%)	45 (78.95%)	35 (53.03%)	63 (67.02%)
Perforating artery	30 (38.46%)	21 (14.19%)	19 (33.33%)	36 (54.55%)	25 (26.6%)
Associated aneurysm	14 (17.95%)	20 (13.51%)	12 (21.05%)	6 (9.09%)	16 (17.02%)
Diffuse nidus	28 (35.90%)	38 (25.68%)	15 (26.32%)	10 (15.15%)	18 (19.15%)
Draining veins					
Stenosis	7 (8.97%)	22 (14.86%)	6 (10.53%)	6 (9.09%)	11 (11.7%)
Any deep drainage	24 (30.77%)	30 (20.27%)	24 (42.11%)	34 (51.52%)	35 (37.23%)
Venous aneurysm	29 (37.18%)	45 (30.41%)	17 (29.82%)	20 (30.3%)	23 (24.47%)
AVM largest diameter(mm)	41.98 (29.71,53.41)	36.70 (27.87,45.45)	44.40 (32.45,60.00)	30.03 (20.01,45.39)	35.39 (26.12,44.14)
AVM location					
Frontal	17 (21.79%)	44 (29.73%)	8 (14.04%)	14 (21.21%)	25 (26.6%)
Temporal	20 (25.64%)	41 (27.7%)	10 (17.54%)	10 (15.15%)	22 (23.4%)
Parietal	27 (34.62%)	36 (24.32%)	19 (33.33%)	16 (24.24%)	27 (28.72%)
Occipital	26 (33.33%)	58 (39.19%)	25 (43.86%)	11 (16.67%)	39 (41.49%)
Subtentorial location	12 (15.38%)	6 (4.05%)	8 (14.04%)	8 (12.12%)	5 (5.32%)
Eloquent location	45 (57.69%)	70 (47.3%)	33 (57.89%)	48 (72.73%)	46 (48.94%)
Deep location	21 (26.92%)	12 (8.11%)	15 (26.32%)	32 (48.48%)	17 (18.09%)
Spetzler-Martin grade					
I	10 (12.82%)	25 (16.89%)	2 (3.51%)	8 (12.12%)	10 (10.64%)
II	21 (26.92%)	49 (33.11%)	21 (36.84%)	17 (25.76%)	35 (37.23%)
III	28 (35.90%)	59 (39.86%)	17 (29.82%)	24 (36.36%)	34 (36.17%)
IV	14 (17.95%)	13 (8.78%)	8 (14.04%)	11 (16.67%)	12 (12.77%)
V	5 (6.41%)	2 (1.35%)	9 (15.79%)	6 (9.09%)	3 (3.19%)
Headache severity					
Mild	32 (41.03%)	52 (35.14%)	17 (29.82%)	30 (45.45%)	37 (39.36%)
Moderate	39 (50.00%)	79 (53.38%)	34 (59.65%)	31 (46.97%)	43 (45.74%)
Severe	7 (8.97%)	17 (11.49%)	6 (10.53%)	5 (7.58%)	14 (14.89%)
Headache frequency					
Occasional	55 (70.51%)	102 (68.92%)	39 (68.42%)	54 (81.82%)	81 (86.17%)
Frequent	21 (26.92%)	39 (26.35%)	15 (26.32%)	12 (18.18%)	12 (12.77%)
Chronic	2 (2.56%)	7 (4.73%)	3 (5.26%)	0 (0%)	1 (1.06%)

*IQR* Interquartile range, *AVM* Cerebral arteriovenous malformations

**Table 5:** Comparison of headache outcome between conservative treatment group and intervention treatment group before PSM

Interventional strategy	**Conservative treatment**		**Intervention treatment**		**OR (95% CI)**	***P*-value**
	Favorable outcome events	Sample Size	Favorable outcome events	Sample Size		
**Microsurgery**	39 (50.00%)	78	137 (92.57%)	148	12.5 (6.02,27.7)	< 0.001
**Embolization**	39 (50.00%)	78	43 (75.44%)	57	3.07 (1.48,6.65)	0.003
**Stereotactic radiosurgery**	39 (50.00%)	78	61 (92.42%)	66	12.2 (4.79,37.8)	< 0.001
**Multimodality treatment**	39 (50.00%)	78	81 (86.17%)	94	6.23 (2.99,12.99)	< 0.001

The univariate logistic regression analysis was used to compare the OR between groups

Statistical significance (*p* < 0.05)

After PSM (Supplemental Material 2-5), multivariate logistic regression analysis adjusted for intervention treatment, female, occipital location, and supply artery dilatation showed that the microsurgery group (OR 7.27, 95% CI 2.82–18.7 *p* < 0.001), stereotactic radiosurgery group(OR 9.46, 95% CI 2.26–39.6, *p* = 0.002), and multimodality treatment group (OR 8.34 95% CI 2.87–24.3, *p* < 0.001) demonstrate significant headache relief compared to the conservative group. However, there was no significant difference between the embolization group (OR 2.24 95% CI 0.88–5.69, *p* = 0.091) and the conservative group (Table 6).

**Table 6:** Comparison of headache outcome between conservative treatment group and intervention treatment group after PSM

Interventional strategy	Conservative treatment		Intervention treatment		OR (95% CI)^$^	*P*-value^$^	OR (95% CI)^*^	*P*-value^*^
	Favorable outcome events	Sample Size	Favorable outcome events	Sample Size				
**Microsurgery**	30(50.85%)	59	52(88.14%)	59	7.18 (2.94,19.7)	< 0.001	7.27 (2.82,18.7)	< 0.001
**Embolization**	22(53.66%)	41	29(70.73%)	41	2.09 (0.85,5.29)	0.110	2.24 (0.88,5.69)	0.091
**Stereotactic radiosurgery**	15(50.00%)	30	27(90.00%)	30	9.00 (2.49,43.6)	0.002	9.46 (2.26,39.6)	0.002
**Multimodality treatment**	29(54.72%)	53	48(90.57%)	53	7.94 (2.92,25.7)	< 0.001	8.34 (2.87,24.3)	< 0.001

Statistical significance (*p* < 0.05)

^$^The results were calculated using univariable logistic regression after PSM

^*^The results were calculated using multivariable logistic regression adjusting for factors (Intervention treatment, female, occipital, and dilation) after PSM

## Discussion

In this study, female gender, supply artery dilatation, and occipital lobe location were identified as potential risk factors for headaches in AVMs. In addition, microsurgery, stereotactic radiosurgery, and multimodal treatment showed significant benefits in headache relief compared to conservative treatment. This study provides important insights into the risk factors for headaches in AVMs and highlights the potential value of aggressive interventions in improving headache symptoms and quality of life.

Currently, there are no specialized studies directly investigating the role of gender in headaches associated with AVM. However, it is widely acknowledged that gender differences play a significant role in migraine prevalence, particularly concerning sex hormonal levels [14]. Approximately 50% of females with migraines suffer from menstrual-related migraines, potentially linked to fluctuations in estrogen levels [15, 16]. This could imply that in AVMs, gender-specific biological mechanisms may influence the risk of headaches. Further research is needed to confirm this hypothesis.

Africk et al.suggested that the absence of stenosis in the draining veins might be associated with headache symptoms in unruptured AVMs [6]. However, the study's conclusions should be interpreted with caution, as several potential predictors of headache were not adjusted due to the limited sample size (e.g., lack of Bonferroni correction). Unfortunately, our subsequent research with an expanded sample size failed to replicate their results. In our study, dilation of the supply artery was identified as a significant risk factor for headaches associated with AVM. This phenomenon may be attributable to hemodynamic alterations caused by AVM [17]. In AVM, abnormal arteriovenous shunting causes blood to bypass the normal capillary network, thereby reducing blood supply to the surrounding normal brain tissue. Under such circumstances, dilation of the supply artery could further exacerbate local ischemia, triggering headaches [18]. The association between headaches and various cerebrovascular ischemic diseases has been extensively reported [19–21]. Activation of the trigeminovascular system is often considered a plausible explanation for the mechanism of headache in unruptured AVMs [18]. This activation may be associated with stimulation of pain-sensitive structures in the head, such as the meninges, arachnoid, and pial vessels, as well as cerebral arteries and venous sinuses [22]. Dilation of supplying arteries could lead to compression or traction of the surrounding neural tissues, thereby activating the trigeminovascular system. Additionally, vascular dilation may be accompanied by the release of neuropeptides, which can stimulate or amplify pain signals [23, 24]. Future studies are needed to confirm these mechanisms.

Multiple studies have noted the connection between occipital lobe AVMs and headache presentations similar to migraines, [18, 25–27] aligning with our research findings. Francesca Galletti observed that 22.5% of patients initially presented with migraine-like headaches, with a significant correlation between the location of the AVM and migraine-like symptoms, predominantly in the occipital lobe [28]. This suggests a possible link between occipital lobe AVM and the pathophysiological mechanisms of migraines, such as cortical spreading depression [18, 29]. Cortical spreading depression is a wave of neuronal and glial depolarization that slowly propagates across the cortex, typically associated with migraine aura. The phenomenon has been extensively studied and is thought to initiate a cascade of biochemical events that can lead to the activation of trigeminal nerves, which causes pain [30]. According to research by Francesca Galletti, the hemodynamic and structural changes surrounding AVM in the occipital lobe may be involved in initiating and propagating cortical spreading depression, thereby triggering headaches [31].

This study offers critical insights into the effectiveness of different treatments in relieving headache symptoms. compared to conservative treatment, microsurgery, stereotactic radiosurgery, and multimodal treatment demonstrate significant advantages in headache relief. This finding highlights the important role of active interventions in ameliorating headache symptoms. Microsurgery may alleviate symptoms by removing the lesion to reduce pressure on the surrounding tissue as well as restoring normal hemodynamics. Stereotactic radiosurgery treatment may reduce the occurrence of headaches by reducing t the blood flow of the supplying artery and gradually blocking the abnormal vessels to restore normal hemodynamics [32, 33]. Multimodal treatment may achieve relief from headaches by harnessing the synergistic effects of integrating various treatment modalities. On the other hand, embolization therapy did not demonstrate statistically significant advantages in headache relief in this study. This may be attributed to the limitations of embolization therapy, such as incomplete embolization of the supplying arteries or failure to normalize the hemodynamic changes leading to the onset of headaches [34]. This finding suggests that although embolization therapy is a crucial means in the treatment of AVM, more research may be needed to optimize treatment strategies specifically in the context of headache management. In addition, Extensive research underscores the integral and significant role of psychological factors in the development and progression of headaches, particularly states of anxiety and depression [35]. Patients with conservatively managed AVM may face heightened psychological challenges due to the uncertainty and fear of potential hemorrhage associated with their condition. Studies indicate that patients with untreated AVM often experience decreased quality of life across several domains such as sleep, emotional behavior, mobility, social interaction, and alertness, along with compromised social functioning compared to the general population. This persistent state of psychological stress not only affects their quality of life but may also trigger or exacerbate headaches, thereby increasing the incidence of headaches among these patients [36].

Limitations need to be acknowledged in this study. Firstly, our research predominantly included individuals with favorable outcomes and did not encompass patients who experienced headaches following a rupture hemorrhage. This selection bias may limit the generalizability of our findings to all AVMs.Secondly, it employed a retrospective design relying on data collected from medical records and telephone interviews. This approach may entail incomplete information and memory biases, thus posing risks of bias or compromised accuracy in the study outcomes. Additionally, the research lacked detailed descriptions of the duration of each headache episode and specific types of painkillers used. This deficiency hindered a more comprehensive understanding of the relationship between headaches and AVM and restricted our ability to perform in-depth analyses. Furthermore, precise data on the timing of headache symptom improvement following various AVM treatments were also lacking, limiting the study's ability to fully assess treatment efficacy. Thirdly, our study did not evaluate other potential influencing factors, such as genetic predispositions, lifestyle choices, and psychological components, which could play significant roles in the development of headaches associated with AVM. In summary, although our study provides valuable insights into headaches in AVMs, these aforementioned limitations suggest the need for prospective study designs in the future to better control biases and collect more comprehensive data for further validation and strengthening of our study findings.

## Conclusions

This study identified potential risk factors for headache in AVMs and found that microsurgery, stereotactic radiosurgery, and multimodal therapy had significant benefits in headache relief compared to conservative treatment. These findings provide important guidance for clinicians when developing treatment options that can help improve overall treatment outcomes and quality of life for patients.

### Supplementary Information

**Supplementary Material 1.** 

## Data Availability

No datasets were generated or analysed during the current study.
