# Supplementary material for: A comprehensive analysis of patients with cerebral arteriovenous malformation with headache: assessment of risk factors and treatment effectiveness
Source: J Headache Pain. 2024 May 7;25(1):72. doi: 10.1186/s10194-024-01774-7 (PMC11075233; doi:10.1186/s10194-024-01774-7)
Supplement: Supplementary file 1 — Supplementary Material 1. [file 10194_2024_1774_MOESM1_ESM.docx]

**Supplemental Material 1 :**

**Protocol for Data Quality Management**

1. Definition of variables were discussed and unified according to the terminology reporting standards or published paper before the initiation of data collection. Clinical research coordinators (CRCs) and neurosurgery residents were then trained by cerebrovascular neurosurgeons or neuroradiologists with more than 15 years’ working experience. CRCs were responsible for demographic information and follow-up data, and neurosurgery residents for angiographic features. The two parts were blinded to each other to ensure the data collected were not biased by imaging characteristics or clinical outcomes.

2. A standard training dataset with 50 cases were used to check the consistency of data collectors. For those variables or cases with significant interobserver variation, the consensus was reached by either modifying the confusing definitions or retraining the data collectors. Only when the consistency reached 90% can the CRC or the resident allowed to extracting information independently.

3. While recording data, one could ask for help about unsure cases in a discussion group with cerebrovascular neurosurgeons in it, or mark these cases and discuss in weekly meetings.

4. The group leader with more than five years’ working experience randomly spot checks these data biweekly. Investigators would receive training again if their data were of low quality, and these data would be recollected by other investigators.

**Supplemental Material 2 :**

**Balance assessment of the propensity score matching between the microsurgery group and the conservative group.**

**
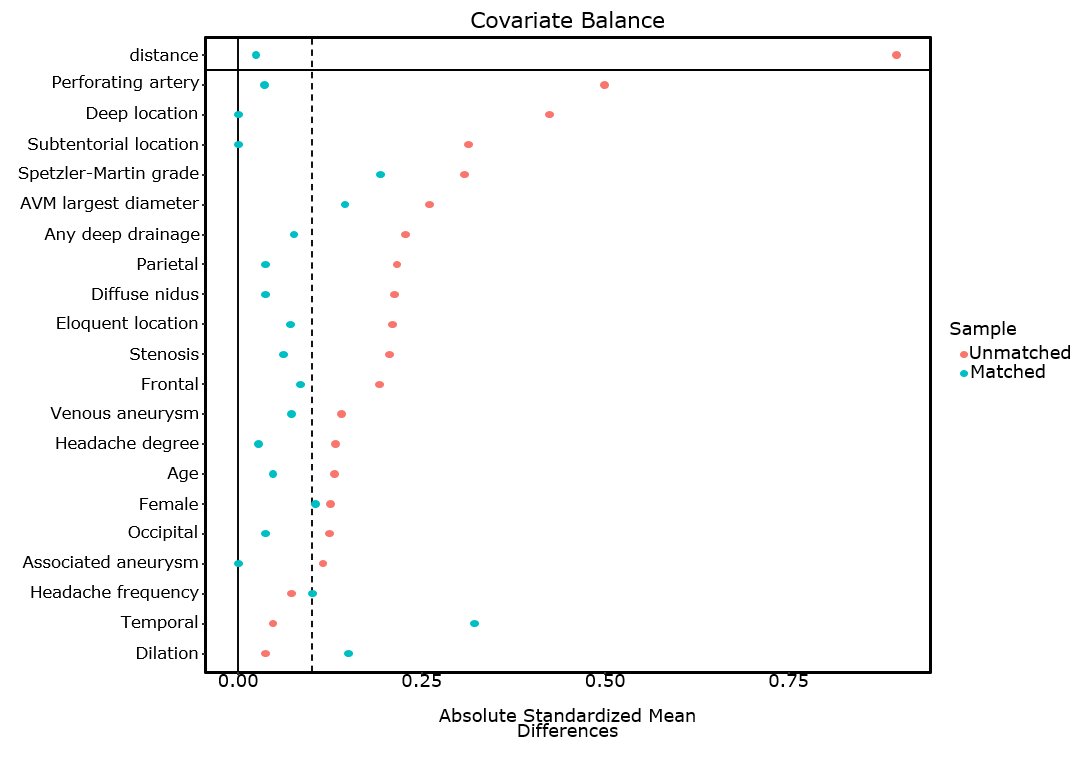
**

**Supplemental Material 3 :**

**Balance assessment of the propensity score matching between the embolization group and the conservative group.**

**
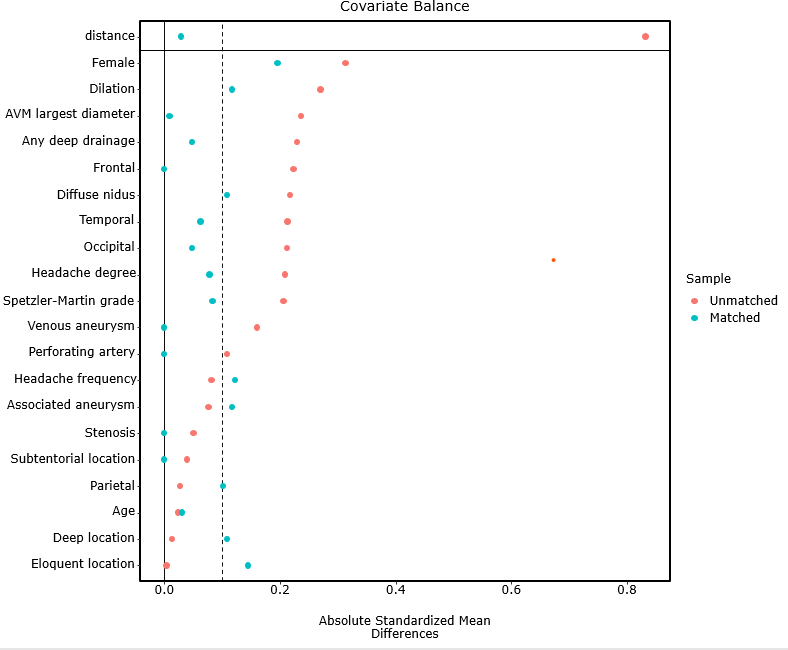
**

**Supplemental Material 4 :**

**Balance assessment of the propensity score matching between the stereotactic radiosurgery group and the conservative group.**

**
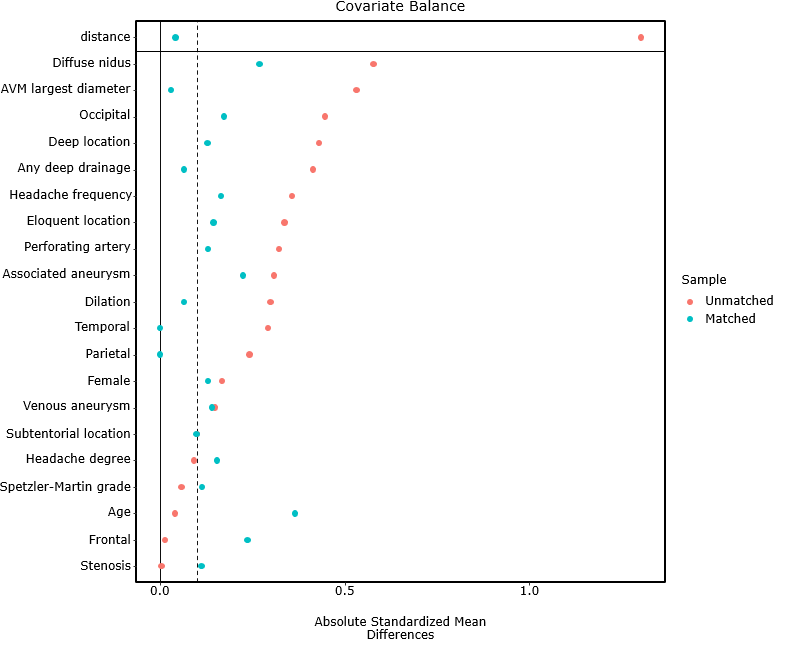
**

**Supplemental Material 5 :**

**Balance assessment of the propensity score matching between the multimodality treatment group and the conservative group.**

**
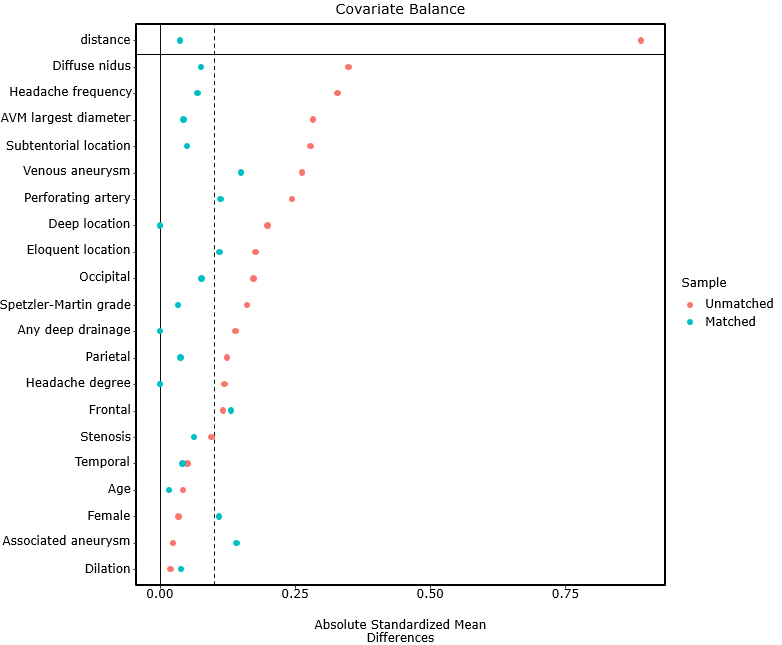
**
